# The Use of Less Conventional Meats or Meat with High pH Can Lead to the Growth of Undesirable Microorganisms during Natural Meat Fermentation

**DOI:** 10.3390/foods9101386

**Published:** 2020-10-01

**Authors:** Christina Charmpi, Emiel Van Reckem, Nikoleta Sameli, David Van der Veken, Luc De Vuyst, Frédéric Leroy

**Affiliations:** Research Group of Industrial Microbiology and Food Biotechnology (IMDO), Faculty of Sciences and Bioengineering Sciences, Vrije Universiteit Brussel, Pleinlaan 2, B-1050 Brussels, Belgium; Christina.Charmpi@vub.be (C.C.); Emiel.Van.Reckem@vub.be (E.V.R.); nikol.sameli@gmail.com (N.S.); David.Van.Der.Veken@vub.be (D.V.d.V.); Luc.De.Vuyst@vub.be (L.D.V.)

**Keywords:** meat fermentation, game meat, enterobacteria, lactic acid bacteria, *staphylococcus*, pH

## Abstract

The bacterial communities that are established during natural meat fermentation depend on the processing conditions and the type of meat substrate used. Six pork samples of variable quality (reflected in pH values) and six less conventional meats (beef, horse, hare, wild deer, wild duck, and wild boar) were naturally fermented under controlled conditions in model systems. The development of lactic acid bacteria (LAB), coagulase-negative staphylococci (CNS), and enterobacteria was followed using culture-dependent techniques and (GTG)_5_-PCR fingerprinting of genomic DNA from the isolates obtained. Taken together, *Latilactobacillus sakei* was the most abundant LAB species, although *Latilactobacillus curvatus* was more manifest in high-pH pork. Within staphylococci, common species were encountered (i.e., *Staphylococcus equorum*, *Staphylococcus saprophyticus*, and *Staphylococcus xylosus*), although some atypical ones (i.e., *Staphylococcus succinus*) were also recovered. Within enterobacteria, *Serratia* spp. prevailed in more acidic pork batches and in beef, whereas *Hafnia* spp. prevailed in game meat fermentations. Enterobacterial counts were particularly high in fermentations with low acidity, namely for some pork batches, hare, wild duck, and wild boar. These findings should be considered when naturally fermented meat products are manufactured, as the use of game meat or meat with high pH can give rise to safety concerns.

## 1. Introduction

Fermented meats, such as fermented sausages, occupy an important place in European food markets [1,2]. Fermented sausages are made from raw meat mixed with fat, curing salt, and spices. They should be considered as the endpoint of a series of complex events, whereby the ingredients are stuffed into casings, fermented, and matured [3,4,5]. In Europe, the use of pork meat is common, followed by beef or horse meat [6,7,8]. In some cases, albeit more rarely, fermented sausages are prepared from game, such as wild boar and deer [9,10,11,12,13,14]. From a microbiological standpoint, this variety presents ample opportunity for investigating the diversity of microbial consortia that can be potentially established during different types of meat fermentation.

In general, microbial communities are variable and difficult to predict, which is especially the case in natural fermentations [15]. Nonetheless, the consortia always consist of lactic acid bacteria (LAB) which drive acidification and coagulase-negative staphylococci (CNS) which contribute to colour and flavour formation [16,17,18,19,20]. Within the group of LAB, *Latilactobacillus sakei* (formerly known as *Lactobacillus sakei*) and, to a lesser degree, *Latilactobacillus curvatus* (formerly known as *Lactobacillus curvatus*) and *Leuconostoc* spp. are most commonly encountered [16,17,18]. Whereas there are usually no specific quality or safety concerns associated with *Latl. sakei* prevalence, *leuconostocs* and *Latl. curvatus* can sometimes produce problematic levels of biogenic amines [19,20]. As far as the CNS communities are concerned, a rather marked species diversity can be encountered, although *Staphylococcus xylosus*, *Staphylococcus saprophyticus*, and *Staphylococcus equorum* are usually the most prevalent species in most natural fermentation processes. Many other CNS species have also been observed, such as *Staphylococcus warneri*, *Staphylococcus epidermidis*, *Staphylococcus sciuri*, and *Staphylococcus succinus* [17,21]. Some CNS species are possibly of concern due to their opportunistic pathogenic nature whereas truly pathogenic coagulase-positive staphylococci may also occur [22]. In a natural fermentation, however, there is no absolute control over which microorganisms are present during fermentation so that food safety risks need to be monitored. Furthermore, it is possible that enterobacteria may be found. Enterobacteria are generally undesired in food fermentations as some species can be pathogenic and/or linked with the presence of biogenic amines [21,23,24].

To mediate unfavourable microbiological consortia, the use of high-quality meat, inoculated with desirable microorganisms under controlled conditions is advisable in view of a safe meat fermentation [16]. A current trend for artisan-type foods and home fermentation is nonetheless discernible, with a renewed interest in natural fermentation [25]. This may lead to the use of less conventional meats, such as game meat. Although this offers an interesting option for product diversification, it could be argued that if the traditional and empirical knowledge that is needed to safely execute such practices is less prevalent, potential risk is created [26]. There has only been a limited amount of studies into the microbiota of game meat, such as wild boar and deer [8,9,12,13,14]. Typically, LAB and CNS communities behave as expected and initial spoilage bacteria tend to decline over time, although more information is needed. Even when using conventional pork mince, variability in the product characteristics may cause fluctuations in product safety and quality [8]. The use of low-quality, dark-firm-dry (DFD) pork meat may cause such fluctuations, which is likely due to its high pH [27]. High pH values are indeed a major modulator of bacterial dynamics during fermentation [15]. 

Given this complex and interlinked fermentation process, there is a need for more integrated and systematic studies of the relationship between meat quality, pH, and microbiota. Therefore, the aim of this study was to chart the bacterial communities that develop during the fermentation of beef, horse, and game meats (i.e., hare, wild deer, wild boar, and wild duck), compared to what occurs during the fermentation of pork mince of variable quality (as reflected in pH) in model systems. The focus was on the major bacterial groups (i.e., LAB, CNS, and enterobacteria), not on the presence of specific pathogens. The research was limited to the effects of the early fermentation stage, being the most critical, and does not account for further potential changes during drying.

## 2. Materials and Methods 

### 2.1. Sample Acquisition

To mimic the fermentation stage of fermented sausage production (excluding the drying stage), a previously developed fermented meat model system was used [15]. To evaluate the impact of the initial pH, a total of six different pork meat batches of different quality were purchased from a local butcher (Brussels, Belgium), according to their colour and exudation level and reflected in their pH value. They were referred to as pork batters 1 to 6. Two batches of raw beef, horse, hare, wild boar, duck, and deer meat were purchased from local supermarkets (Brussels, Belgium). Each meat batter consisted of fresh mince (2 kg, 14% (m/m) fat fraction), sodium nitrate at 150 mg/kg (VWR International, Darmstadt, Germany), ascorbic acid at 500 mg/kg (Sigma-Aldrich, St. Louis, MO, USA), and sodium chloride at 3.0% (m/m; VWR International). 

Each mixture was divided into a total of four plastic 60-mL containers per batch, containing about 60 g per container (closed lid) to fill the volume and permit fermentation in the absence of air. The containers were deposited into an incubator at 23°C. Samples were taken for analysis at the start and after 7 days of fermentation. Previous analysis has indicated that the bacterial diversity in the fermented meat models mentioned above stabilizes at around 3 days of fermentation, which is the usual industrial practice, and does not differ much from the situation after one week. Nonetheless, in the present study, a 7-day sampling point was chosen to maximize the effects of selection pressure and because natural, artisan-type fermentation processes often take several more days [15].

At each time point, two containers were analysed. For the bacterial enumerations, one sample per container was taken (duplicate data). Subsequently, for pH and water activity (a_w_) measurements, three measurements were performed (see below).

### 2.2. Enumeration and Isolation of Microorganisms

Colony enumeration and isolation were performed as described previously [23]. Some 10–15 g of meat sample was aseptically transferred into a stomacher bag (Seward, Worthing, West 99 Sussex, UK), with the addition of recovery diluents in ratio 1:10 (depending on the weight of the meat sample) (sterile solution of 0.85% (*m*/*v*) NaCl (VWR International) and 0.1% (*m*/*v*) bacteriological peptone (Oxoid, Basingstoke, Hampshire, UK)). This mixture was homogenized at maximum speed for 2 min in a Laboratory Blender Stomacher 400 (Seward) and appropriate decimal dilutions in saline (0.85% (*m*/*v*) NaCl) were prepared and spread on three selective agar media. Mannitol salt-phenol red-agar (MSA; VWR International), de Man-Rogosa-Sharpe (MRS) agar (Oxoid), and RAPID’Enterobacteriaceae agar (RAPID’Entero agar; Bio-Rad laboratories, Hercules, CA, USA) were used for the enumeration of presumptive catalase-positive cocci (in particular CNS), LAB, and enterobacteria, respectively. The MSA and MRS agar media were incubated at 30 °C for 72 h and the RAPID’Entero agar medium at 30 °C for 24 h. MSA, MRS agar, and RAPID’Entero agar media containing 30 to 300 colonies were retained to pick colonies (20–30%) to follow the bacterial community dynamics. These colonies were randomly selected and transferred into brain heart infusion (BHI; Oxoid). The cultures were incubated overnight at 30 °C and used for DNA extraction as well as for storage at −80 °C in cryovials containing 25% (*v*/*v*) of glycerol. 

### 2.3. pH and a_w_ Measurements

The pH of the meat batter was measured with a DY-P10 pH meter (Sartorius, Göttingen, Germany) equipped with an insertion pH probe (VWR International, Darmstadt, Germany). The *a*_w_ was measured at 25 °C with a Hydropalm 23 *a*_w_ meter (Rotronic, New York, NY, USA). Three independent measurements were performed per sample.

### 2.4. Classification and Identification of Bacterial Isolates through (GTG)_5_-PCR Fingerprinting of Genomic DNA

Genomic DNA extraction from cell pellets obtained by microcentrifugation at 13,000 rpm of 1.5 mL of an overnight culture of the isolates mentioned above was performed with a Nucleospin 96 tissue kit (Macherey Nagel, Düren, Germany), according to the manufacturer’s instructions. Prior to extraction, all cell pellets were washed with Tris-ethylene diaminetetraacetic acid (EDTA)-sucrose buffer (TES buffer; 50 mM Tris base (Calbiochem, Darmstadt, Germany), 1 mM EDTA (Sigma-Aldrich), and 6.7% (*m*/*v*) sucrose (VWR International); pH 8.0). Subsequently, (GTG)_5_-PCR fingerprints of the genomic DNA were obtained that were subjected to image analysis, as described previously [28]. Numerical cluster analysis of the fingerprints obtained was performed with the BioNumerics 5.1 software (Applied Maths, Sint-Martens-Latem, Belgium). The clustering of the fingerprints was calculated based on the Pearson product-moment correlation coefficient. Dendrograms were then generated with the unweighted pair group method with arithmetic average (UPGMA) clustering algorithm. To confirm the species identity assigned to each cluster, random isolates were selected and identified by sequencing the 16S rRNA gene and/or *rpoA*, *rpoB*, and *tuf* genes, as described previously [29,30]. For the molecular identification of *Latl. sakei* and *Latl. curvatus*, the reverse primers Ls (5′-ATG AAA CTA TTA AAT TGG TAC-3′) and Lc (5′-TTG GTA CTA TTT AAT TCT TAG-3′), coupled with the forward primer 16S (5′-GCT GGA TCA CCT CCT TTC-3′), were used [31]. The amplification conditions were as described by these authors. 

### 2.5. Statistics

Intrasample diversity (alpha-diversity) was assessed by calculating the Simpson (diversity) and Pielou (evenness) indexes. To identify discrete bacterial communities between the samples and to quantify the effect of the different meat types on the bacterial communities within these fermentation processes, a permutational analysis of variance (PERMANOVA) based on the Bray–Curtis dissimilarity matrix was performed. This analysis was followed by a similarity percentage analysis (SIMPER) to assess the bacterial differences between meat types. Further, Pearson correlation coefficients were calculated to test the effect of pH on the bacterial counts and relative abundances of the species found using the Hmisc package (version 4.4-0) [32]. The packages vegan, (version 2.5-5) [33], and RVAideMemoire, (version 0.9-73) [34], were implemented for both intrasample and intersample variability assessment. All samples were also subjected to a principal component analysis (PCA) to explore the isolate identification data. PCA was performed using vegan and visualized by using the package ggplot2 (version 3.1.1) [35]. Subsequently, one-way analysis of variance (ANOVA) was conducted for the determination of differences in bacterial enumerations and of pH between all fermentation samples, followed by a series of post-hoc pairwise comparisons with Tukey’s test. A threshold value of 0.05 was considered to be significant for all statistical procedures applied. All statistical analyses and tests performed were executed through the RStudio software (version 3.5.2) [36]. 

## 3. Results

### 3.1. The Effect of Meat Quality as Reflected in Initial pH Values on the Bacterial Community Dynamics during Fermentation of Pork Mince

The various raw pork minces were analysed with respect to their pH and *a*_w_ values. The initial pH of the sausage pork batter varied from 6.02 ± 0.01 to 5.62 ± 0.01 and decreased after 7 days of fermentation to values from varied from 5.90 ± 0.02 to 5.21 ± 0.01, respectively (Table 1). The variability of pH was reflected in significance difference between the samples, both at day 0 and 7 (Table 1). Moreover, the drop in pH of the pork meat samples with the highest initial acidity (pork batters 5 and 6) was significantly higher than for the rest of the pork meat samples (*p* < 0.05). The initial a_w_ was on average 0.982 ± 0.003 but decreased as fermentation proceeded over time (Table S1).

Presumptive LAB populations, as measured on MRS agar media, ranged from 4.19 ± 0.50 log (cfu/g) to 5.76 ± 0.60 log (cfu/g) in the initial pork meat batters and increased to levels between 7.91 ± 0.20 log (cfu/g) and 8.84 ± 0.40 log (cfu/g) after 7 days of fermentation (Table 1). Staphylococcal counts, as measured on MSA media, were initially situated between 3.70 ± 0.30 log (cfu/g) and 4.57 ± 0.50 log (cfu/g). After 7 days of fermentation, they increased in numbers that varied from 5.40 ± 1.00 log (cfu/g) to 6.37 ± 0.80 log (cfu/g). Lastly, presumable enterobacterial populations, derived from the counts on RAPID’Entero agar media, equalled 3.75 ± 0.50 log (cfu/g) to 4.02 ± 0.10 log (cfu/g) initially (Table 1). At low pH values (samples of pork batters 5 and 6), the counts of presumable enterobacterial populations remained stable during fermentation. At high pH values, however, presumable enterobacterial populations increased over time. The counts on RAPID’Entero agar media of meat batters with the highest end-pH values (samples of pork batters 1 and 2) were significantly higher than those in samples of pork batters 3–6 by the end of the experiment (*p* < 0.05). This was not the case for the counts on MRS agar (*p* = 0.92) and MSA media (*p* = 0.84).

As mentioned above, biodiversity was assessed by using (GTG)_5_-PCR fingerprinting. The accession numbers of the representative isolates identified, as well as their percentages of sequence identity, are listed in Table 2. 

**Figure 1:**
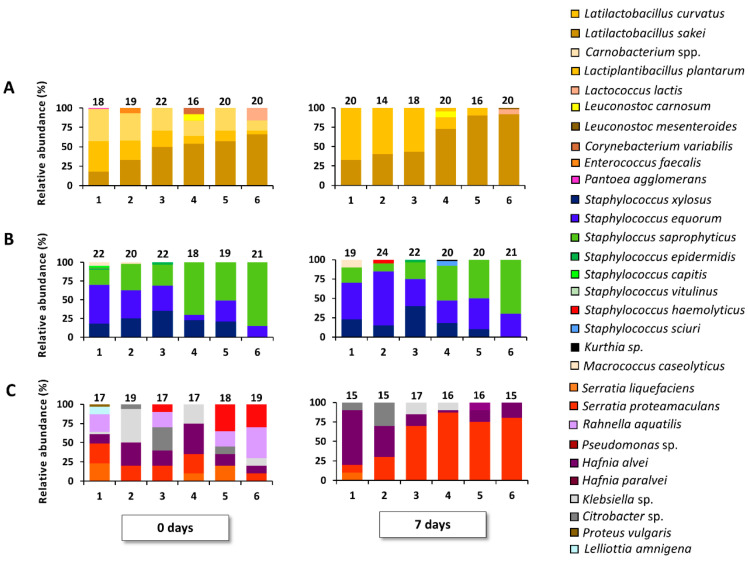
Course of bacterial species isolated from MRS agar (**A**), MSA (**B**), and RAPID′Entero agar (**C**) of pork batters 1, 2, 3, 4, 5, and 6, fermented at 23 °C for 7 days. Τhe number of isolates for identification is mentioned above each bar. The bacterial species diversity is displayed as relative abundances, calculated based on the number of isolates (N) obtained per time point (0 and 7 days of meat fermentation).

Regarding LAB, the following species were isolated during the pork meat fermentations: *Latl. sakei*, *Latl. curvatus*, *Lactococcus lactis*, *Carnobacterium* spp., *Leuconostoc carnosum*, and *Enterococcus* sp. Initially, LAB communities consisted mainly of *Latl. sakei*, *Latl. curvatus*, and carnobacteria. However, carnobacteria vanished by the end of the fermentation processes (Figure 1, Table S2). Additionally, a consistent prevalence shift from *Latl. sakei* to *Latl. curvatus* was seen over the pH spectrum of the various samples, both at the start and after 7 days of fermentation. A strong correlation between *Latl. sakei* and the acidification level was found (*r* = −0.94, *p* < 0.05). 

Within the species diversity recovered from MSA media, a coprevalence of *S. xylosus*, *S. equorum*, and *S. saprophyticus* was found (Figure 1; Table S3). In addition, *S. epidermidis*, *S. vitulinus*, *Staphylococcus capitis*, *S. pasteuri*, *Staphylococcus haemolyticus*, *Kurthia* sp., and *Macrococcus caseolyticus* were present as minor species. The most striking trend was that S. saprophyticus became increasingly manifest as pH values decreased across the samples.

With respect to the RAPID’Entero agar isolates, a high species diversity was seen at the start of the fermentation processes (Figure 1; Table S4). The initial communities were composed of *Serratia proteamaculans*, *Serratia liquefaciens*, *Hafnia alvei*, *Hafnia paralvei*, *Rahnella aquatilis*, *Klebsiella* sp., *Citrobacter* sp., and *Proteus vulgaris*. After 7 days of fermentation, however, *Serratia* spp. took over at the low pH values (Figure 1; Table S4). A Pearson correlation analysis revealed that *Serratia* spp. and pH were significantly correlated (*r* = −0.72, *p* < 0.05).

### 3.2. Alpha- and Beta-Diversity of the Pork Mince Fermentation Processes

**Figure 2:**
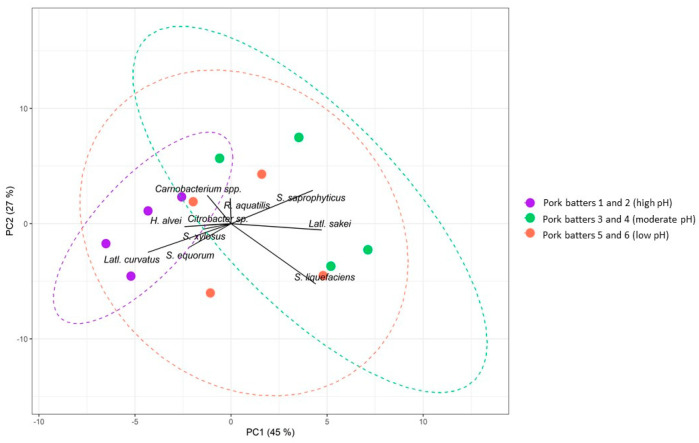
Centred principal component analysis (PCA) biplot based on Bray–Curtis dissimilarity scores of the bacterial community structures, with factor loadings, of pork fermentation processes. Only factor loadings with an absolute value above 1 were considered. Colours indicate different sample groups characterized by high pH (purple), moderate pH (orange), or low pH (green). The proportion of variance for every PC is indicated between brackets. The dotted lines represent 90% normal confidence ellipses for the sample groups.

In general, the bacterial diversity of all pork-based meat fermentation processes was high (Table S5). When differentiating samples according to high (samples of pork batters 1 and 2), moderate (samples of pork batters 3 and 4), and low pH (samples of pork batters 5 and 6), a significant impact was seen on the bacterial species diversity (*p* < 0.05), based on a PERMANOVA. These differences in community compositions of the different samples were supported by PCA (Figure 2). This also underpinned the distinction between high-pH and low-pH samples based on their bacterial community structures. Bacterial profiles in samples from the meat batters with high initial pH were significantly different (*p* < 0.05) from those at moderate and low initial pH. SIMPER analysis confirmed that the bacterial differentiation between the fermentation processes of high-, moderate-, and low-pH pork meat could be attributed to differences in the following bacterial species present: *Latl. sakei*, *Latl. curvatus*, *carnobacteria*, *S. saprophyticus*, *S. equorum*, *S. liquefaciens*, and *H. alvei*. 

### 3.3. The Effect of Less Conventional Meat Types on the Bacterial Community Dynamics during Fermentation

The initial a_w_ of the meat samples did not vary much between the different meat types and was on average 0.981 ± 0.003 initially and 0.966 ± 0.001 after 7 days of fermentation (Table S6). In contrast, a correlation between pH and the meat type was found (*p* < 0.05), and variations in pH were found according to the meat type, albeit not always significant (Table 3). Fermentations of hare, wild boar, and wild duck were characterized by higher pH values than was the case for beef, horse, and wild deer, although not significant (*p* = 0.15). 

Initial counts on MRS agar for hare, wild boar, and wild duck fermentation processes were higher (6.49 ± 0.46 log (cfu/g), on average) than for beef, horse, and wild deer (4.84 ± 0.55 log (cfu/g), on average), although not significant (*p* = 0.86) (Table 3). These differences were not encountered at the end of the fermentation processes, as the final counts on MRS agar for the duplicate fermentations for each meat type converged, averaging 8.39 ± 0.49 log (cfu/g) overall. The counts on MSA at the onset of the fermentation processes ranged from 3.26 ± 0.30 to 4.40 ± 0.55 log (cfu/g), and from 4.78 ± 0.08 to 6.03 ± 0.20 log (cfu/g) after 7 days of fermentation (Table 3). Notably, counts on MSA for hare fermentation samples strongly decreased during the fermentation process. In contrast, counts on RAPID’Entero agar differed between fermentation samples. For hare, wild boar, and wild duck fermentation processes, counts averaged 6.65 ± 1.06 log (cfu/g) at the beginning and 6.30 ± 0.86 log (cfu/g) after 7 days of fermentation. For the beef, horse, and wild deer fermentation processes, RAPID’Entero agar counts were generally lower, averaging 4.15 ± 0.16 log (cfu/g) at the onset of fermentation and 2.81 ± 0.18 log (cfu/g) after 7 days of fermentation (Table 3). 

The LAB community profiles of all fermentation samples were similar (Table S7). They were characterized by a high prevalence of *Latl. sakei*, whereas *carnobacteria* and *Latl. curvatus* were retrieved at the beginning of the fermentation processes but vanished over time. Other bacterial species that were only occasionally isolated were *Leuconostoc* spp., *Carnobacterium variabilis*, *Enterococcus faecium*, *Klebsiella* sp., and *H. alvei*. 

**Figure 3:**
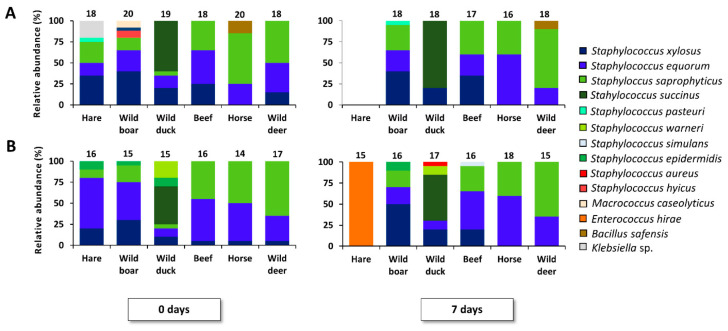
Course of bacterial species isolated from MSA during fermentation of less conventional meat types, including two replicates (**A**,**B**) at 23 °C for 7 days. Τhe number of isolates for identification is mentioned above each bar. The bacterial species diversity is displayed as relative abundances, calculated based on the number of isolates (N) obtained per time point (0 and 7 days of meat fermentation).

Within the MSA media isolates, more species diversity was obtained (Figure 3, Table S8). A coprevalence of *S. saprophyticus*, *S. xylosus*, and *S. equorum* was found in all cases, except for the hare and duck fermentation processes. An increasing relative abundance of *S. saprophyticus* was found in the fermentation samples that were more acidic (i.e., the beef, horse, and wild deer fermentation processes), as its presence also correlated with the pH of the meat batter (*r* = −0.47, *p* < 0.05). One replicate of the hare fermentation processes did not allow for recovery of MSA media isolates, whereas the other replicate resulted in the isolation of *Enterococcus hirae* at day 7. In the fermented duck samples, the presence of *S. succinus* stood out. Lastly, the pathogens *Staphylococcus aureus* and *Staphylococcus hyicus* were found in one replicate of the wild boar and wild duck fermentations, in very low abundance (Table S8).

The enterobacterial profiles were variable. The main species isolated belonged to the *Hafnia* and *Serratia* genera, followed by *R. aquatilis*, *Klebsiella* sp., and *Citrobacter* sp. The species *R. aquatilis* was mainly present at the onset of the fermentation processes (Table S9). In general, a high prevalence of *Hafnia* spp. was found after 7 days of fermentation for all meat types, except for the beef fermentation processes, in which *Serratia* spp. prevailed.

### 3.4. Alpha- and Beta-Diversity of the Less Conventional Meat Fermentation Processes

The bacterial diversity of all meat samples investigated was relatively high, except for the hare fermentation processes that were characterized by a more narrow species diversity (Table S10). As mentioned above, this was related to the absence of isolates in one replicate and the high prevalence of *E. hirae* in the other. 

The meat fermentation samples were statistically different (*p* < 0.05), indicating differences in their bacterial community structures. Pairwise comparison revealed that the bacterial profiles in the beef fermentation samples were different from those in the wild boar, wild duck, horse, and wild deer ones (*p* < 0.05). In turn, horse fermentation samples differed from those of wild boar and wild duck fermentation processes (*p* < 0.05) and wild boar fermentation samples were different from those of wild duck and deer ones (*p* < 0.05). Wild deer fermentation samples were different from those of hare and wild duck fermentation processes (*p* < 0.05) and hare fermentation samples from those of wild duck ones (*p* < 0.05). 

**Figure 4:**
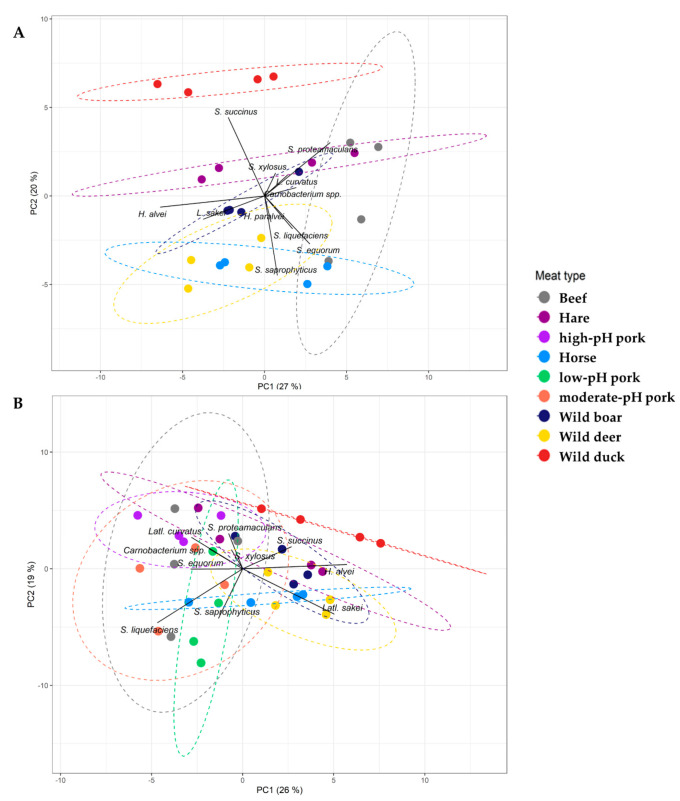
Centred principal component analysis (PCA) biplot based on Bray–Curtis dissimilarity scores of the bacterial community structures obtained during fermentation of less conventional meat types (**A**) and extended with the fermented pork samples to the entire dataset (**B**), with factor loadings. Only factor loadings with an absolute value above 1 were considered. Colours indicate different sample groups, beef (gray), hare (dark purple), high-pH pork (light purple), horse (light blue), low-pH pork (green), moderate-pH pork (orange), wild boar (dark blue), wild deer (yellow), and wild duck (red). The proportion of variance for every PC is indicated between brackets. The dotted lines represent 90% normal confidence ellipses for the sample groups.

To explore differences in beta-diversity, a PCA was performed (Figure 4A). Fermentation samples from different meat types clustered closely, because of similarities in their bacterial community structures. However, certain differences in the course of the bacterial communities were obtained. SIMPER analysis showed that these differences could be attributed to the characteristic prevalence of *S. succinus* in duck fermentation processes, abundance of *S. saprophyticus* in those samples with low pH values (i.e., beef, horse, and wild deer), and prevalence of *Serratia* spp. in beef fermentation samples (in contrast to *Hafnia* spp. in the fermentation samples of the other meat types).

### 3.5. Beta-Diversity of the Entire Dataset of Meat Types

When analysing all the above-mentioned meat type fermentation samples within one dataset, thus including both the pork and less-conventional meats, it was uncovered that the meat type had a significant impact on the RAPID’Entero agar counts (*p* < 0.05) but not on the MRS agar (*p* = 0.08) and MSA counts (*p* = 0.07). The pH values and RAPID’Entero agar counts were correlated significantly (*r* = 0.72, *p* < 0.05) but this was, once more, not the case for the counts on MRS agar (*p* = 0.21) and MSA (*p* = 0.12). The meat type also had a significant impact on the bacterial species diversity (*p* < 0.05). A negative correlation was found between pH and *Latl. sakei* (*r* = −0.5, *p* <0.05), whereas a positive correlation was found between pH and *Latl. curvatus* (*r* = 0.37, *p* < 0.05). The pH and *S. saprophyticus* were negatively correlated (*r* = −0.41, *p* < 0.05). Pairwise comparisons revealed that the bacterial profiles in the wild duck fermentation samples were different from all the others and additionally, that the fermentation samples of low-pH pork differed from those of high-pH pork and hare ones (*p* < 0.05) (Table 4). Finally, to visualize these differences in beta-diversity of the isolate identification data, a PCA was performed (Figure 4B), in which samples with correlative bacterial community structures clustered closely.

## 4. Discussion

Although meat fermentation normally narrows the initial microbiota down to a desirable consortium, this can be unsuccessful at times due to contaminated raw material or improper processing conditions, such as poor acidification [11,37]. In some cases, traditional fermented meat products are still obtained by natural fermentation. This may pose certain risks, especially when deviating from conventional practice and traditional knowledge [38]. As outlined further below, the present study revealed that natural meat fermentation processes may indeed lead to the development of potentially worrisome bacteria, especially when the raw meat has a high initial pH. Acidification seems to be crucial for a safe fermentation. Enterobacteria are relatively acid-sensitive and are quickly outcompeted during food fermentations that have an acidifying profile [39]. In addition, in the case of game meat, the microbiological safety can be compromised because of a higher chance of the presence of undesirable bacteria in the raw meat [40,41]. This may be due to contamination during hunting or to the fact that game meat is often characterized by high pH values [12,14]. 

In all meat fermentation processes studied, LAB were the prevalent species and the major drivers of fermentation. High abundance of *Latl. sakei* usually serves as a reassuring finding, as prevalence of this benign species is the usual state of affairs in well-performed meat fermentation processes [17,18,42,43,44]. In the present study, *Latl. sakei* was the most frequently isolated LAB species when using pork, as long as the pH was low enough. In the less acidified pork fermentation samples, however, *Latl. curvatus* became more persistent, as this species is known to prefer a higher pH than *Latl. sakei* [18,27]. This can be problematic since *Latl. curvatus* can be a major producer of biogenic amines [45]. High concentrations of biogenic amines pose a health risk, since they are neurotoxic, and they have been linked with food poisoning [46]. Higher abundance of *Latl. curvatus* in low-acidified pork batters indicates that, even in habitual natural pork fermentation processes, potentially undesirable microorganisms can prevail if the raw material or processing conditions deviate in certain key characteristics, such as acidification levels.

This trend was further evidenced in the CNS communities. A prevalence of *S. xylosus*, *S. saprophyticus*, and *S. equorum* was found in most meat fermentation processes, as is common in natural meat fermentation processes [42,47,48,49]. Yet, low acidity allowed the proliferation of several other CNS species as subdominant ones, among which *S. epidermidis*. The latter species is an opportunistic pathogen and a causal agent in (skin) infections [15]. It was present in low abundance in the meat batters with pH values above 5.8, as it is less competitive in acidic environments [50]. In one poorly acidified pork fermentation sample, the opportunistic pathogen *Staphylococcus haemolyticus* was encountered. This species is indeed able to appear during meat fermentation processes [51], although it thrives poorly under proper acidification conditions [52]. 

The present study showed that the use of nonconventional meat types needs to be carefully thought through, as several potentially pathogenic bacteria emerged during meat fermentation, likely due to the concomitant high-pH levels (even if this was not the focus of the study). In wild boar and duck fermentation processes, characterized by a relatively high initial pH, the pathogens *S. hyicus* and *S. aureus* were isolated, albeit at low levels, which were in accordance with previous findings [53,54,55], and do not inevitably pose a threat for enterotoxin production [56]. This nonetheless leads to potential risk and deserves attention [57]. In hare fermentation processes, no CNS species were retrieved, but *E. hirae* was isolated from MSA. This species has not only been associated with rabbit, wild rabbit, and hare faeces before, but its presence is also linked with urinary infections in humans [58,59,60]. In wild duck fermentation processes, *S. succinus* was the main species isolated. To our knowledge, this is the first time *S. succinus* has been reported in duck meat fermentation processes, although it has been found in other fermented meat products before [42,61]. This is not necessarily a matter of concern, but some strains of *S. succinus* exhibit proteolytic, lipolytic, and urease activities and can also be hemolytic and toxigenic [62,63]. Further, one should be mindful of the shortcomings and limitations of culture-based methods as they cannot identify nonculturable cells [64].

Enterobacterial counts generally showed an increase during meat fermentation processes characterized by high pH values, as such conditions favour their growth [56,65]. At the end of the fermentation processes with beef or low-pH pork, *Serratia* spp. prevailed within the enterobacterial group. This species has indeed a high degree of adaptation to more acidic meat environments [66]. *Hafnia* spp. prevailed in the nonconventional meat fermentation processes. Both *Hafnia* spp. and *Serratia* spp. are known putrescine and cadaverine producers and their presence is associated with the quality defect of green discolouration in meat too [67,68]. 

## 5. Conclusions

The present study evaluated the bacterial diversity of a variety of conventional and game meat fermentation processes, which contributes to a deeper understanding of how the meat type and acidification level influence the bacterial composition. A high starting pH in pork and other meat types used for natural fermentation processes allowed the proliferation of problematic bacteria. Hence, the use of good quality meat is of importance and particularly so for game meat fermentation processes, as such fermentation processes have not been all that well characterized yet with respect to their microbiology. Further care should be taken with natural fermentation of high-pH pork meat or with hare, wild boar, and wild duck meat. Proper acidification of the meat batter is crucial and allows the raw meat to be transformed into a safe end product. The findings obtained in the current study can be further explored by investigating the microbial diversity during actual dry-fermented sausage production on pilot scale. In time, knowledge gathered from such studies would strengthen our understanding of the impact of initial pH and different meat types on microbial ecology.

## Figures and Tables

**Table 1:** Community dynamics of the microbiota of naturally fermented pork mince and the resulting pH course after 0 and 7 days of fermentation and standard deviation (SD). The bacterial counts on de Man-Rogosa-Sharpe (MRS) agar, mannitol salt-phenol red-agar (MSA), and RAPID’Enterobacteriaceae agar (RAPID′Entero agar) are expressed as log (cfu/g). Data sets with superscripts were significantly heterogeneous according to ANOVA; within each sampling time, means sharing the same superscript were not significantly different from each other using post-hoc tests.

Time (days)	Sample	pH	SD	MRS Agar Counts (log(cfu/g))	SD	MSA Counts (log(cfu/g))	SD	RAPID’Entero Agar Counts (log(cfu/g))	SD
0	1	6.02 ^a^	0.01	5.76	0.60	3.70	0.30	3.93	0.26
	2	5.99 ^a^	0.02	5.12	0.36	3.90	0.10	4.02	0.10
	3	5.85 ^a,b^	0.01	4.19	0.50	4.32	0.28	3.86	0.18
	4	5.80 ^a,b^	0.01	4.27	0.45	3.89	0.15	3.75	0.50
	5	5.72 ^b^	0.01	4.6	0.30	3.81	0.11	3.00	0.30
	6	5.62 ^b^	0.01	4.97	0.45	4.57	0.50	2.77	0.15
7	1	5.90 ^a^	0.01	8.27	0.25	5.40	0.22	5.80 ^a^	0.41
	2	5.78 ^a^	0.01	8.33	0.18	5.85	0.25	5.33 ^a^	0.22
	3	5.65 ^a^	0.01	8.47	0.22	6.37	0.46	3.79 ^b^	0.42
	4	5.56 ^a^	0.01	8.12	0.15	5.43	0.20	3.90 ^b^	0.21
	5	5.24 ^b^	0.01	7.91	0.20	5.68	0.32	3.10 ^b^	0.45
	6	5.21 ^b^	0.01	8.84	0.71	5.80	0.64	2.60 ^b^	0.33

**Table 2:** Identities of the 16S rRNA, and/or *rpoB*, *tuf*, and *rpoA* genes sequenced from genomic DNA of representative bacterial isolates picked from MRS agar, MSA, and RAPID’Entero agar, and the accession numbers of the entries with the highest identity.

Bacterial Species	Gene	Identity (%)	Accession Number
*Carnobacterium* spp.	16S rRNA	99	NR_113798.1, NR_042093.1
*Latilactobacillus curvatus*	16S rRNA	100	NR_114915.1
*Latilactobacillus sakei*	16S rRNA	100	NR_113821.1, NR_115172.1
*Lactiplantibacillus plantarum*	16S rRNA	100	NR_104573.1
*Lactococcus lactis*	16S rRNA	100	NR_113925.1
*Leuconostoc carnosum*	16S rRNA	100	NR_040811.1
*Leuconostoc mesenteroides*	16S rRNA	99	NR_157602.1
*Weissella fabalis*	16S rRNA	98	NR_108858.1
*Brochothrix thermosphacta*	16S rRNA	100	NR_113587.1
*Macrococcus caseolyticus*	16S rRNA, *tuf*	98	NR_119262.1, AP009484.1
*Corynebacterium variabilis*	16S rRNA	100	NR_025314.1
*Enterococcus faecium*	*rpoB*	100	CP021885.1, NR_115764.1
*Enterococcus hirae*	*rpoB*, *tuf*	100	CP003504.1, CP023011.2
*Staphylococcus xylosus*	*rpoB*, *tuf*	99	CP008724.1, CP031275.1
*Staphylococcus equorum*	*rpoB*	100	CP013980.1
*Staphylococcus saprophyticus*	*rpoB*	100	CP022093.2, CP014113.2
*Staphylococcus epidermidis*	*rpoB*	99	CP009046.1
*Staphylococcus vitulinus*	*rpoB*, *tuf*	100	HM352960.1, KY011914.1
*Staphylococcus capitis*	*rpoB*	99	CP007601.1
*Staphylococcus haemolyticus*	*rpoB*	99	CP013911.1
*Staphylococcus succinus*	*rpoB*	100	CP018199.1
*Staphylococcus pasteuri*	*tuf*	99	CP017463.1
*Staphylococcus hyicus*	*rpoB*	99	CP008747.1
*Staphylococcus aureus*	*rpoB*	99	AP017922.1
*Bacillus safensis*	*rpoB*	100	CP018197.1
*Serratia proteamaculans*	*rpoA*	100	CP000826.1
*Serratia liquefaciens*	*rpoA*	100	CP033893.1, CP014017.2
*Hafnia alvei*	*rpoA*	100	CP015379.1
*Hafnia paralvei*	*rpoA*	99	CP014031.2
*Rahnella aquatilis*	*rpoA*	99	CP003244.1
*Lelliotia amnigena*	*rpoA*	99	CP015774.2
*Citrobacter* sp.	*rpoA*	99	CP022049.2
*Proteus bulgari*	*rpoA*	100	CP033736.1
*Enterobacter* sp.	*rpoA*	100	CP041062.1
*Klebsiella* sp.	*rpoA*	99	CP011077.1
*Pantoea agglomerans*	*rpoA*	99	CP016889.1
*Pseudomonas* sp.	16S rRNA	99	NR_148763.1
*Kurthia* sp.	16S rRNA	100	NR_118296.1

**Table 3:** Community dynamics of the microbiota of various naturally fermented, less conventional meat types and the resulting pH course after 0 and 7 days of fermentation, displayed as average of two biological replicates carried out for each meat type, and standard deviation (SD). The bacterial counts on MRS agar, MSA, and RAPID′Entero agar are expressed as log (cfu/g).

Time (days)	Sample	pH	SD	MRS Agar Counts (log(cfu/g))	SD	MSA Counts (log(cfu/g))	SD	RAPID’Entero Agar Counts	SD
0	Hare	5.94	0.15	5.28	0.59	3.26	0.33	5.55	0.80
	Wild duck	5.84	0.12	7.56	0.75	3.78	0.37	7.67	1.43
	Wild boar	5.82	0.02	6.64	0.07	3.84	0.50	6.72	1.74
	Beef	5.67	0.06	4.28	0.08	3.97	0.04	4.08	0.01
	Horse	5.54	0.03	4.65	0.98	4.40	0.39	4.33	0.29
	Wild deer	5.51	0.03	5.59	0.35	3.86	0.37	4.03	0.13
7	Hare	5.52	0.08	8.79	0.06	1.76	2.53	5.41	0.55
	Wild duck	5.96	0.26	9.07	0.24	4.78	1.57	7.12	2.84
	Wild boar	5.95	0.75	8.68	0.12	5.66	0.04	6.36	3.21
	Beef	5.27	0.17	7.72	0.55	6.03	0.91	2.68	0.46
	Horse	5.05	0.04	8.20	0.10	4.89	0.06	3.01	0.02
	Wild deer	5.10	0.01	7.88	0.54	4.90	0.08	2.73	0.42

**Table 4:** P values based on pairwise comparisons between all meat fermentation samples using PERMANOVA on a distance matrix.

Meat Type	1	2	3	4	5	6	7	8	9
1. Beef									
2. Hare	0.079								
3. High-pH pork	0.047	0.079							
4. Horse	0.047	0.105	0.047						
5. Low-pH pork	0.183	0.047	0.047	0.238					
6. Moderate-pH pork	0.130	0.079	0.105	0.047	0.310				
7. Wild boar	0.047	0.183	0.047	0.047	0.047	0.047			
8. Wild deer	0.047	0.047	0.047	0.183	0.047	0.047	0.047		
9. Wild duck	0.047	0.047	0.047	0.047	0.047	0.047	0.047	0.047	

## Supplementary Materials

The following are available online at https://www.mdpi.com/2304-8158/9/10/1386/s1, Table S1. Water activity (*a*_w_) values of the pork mince fermentation samples of pork batters 1, 2, 3, 4, 5, and 6, incubated at 23 °C for 0 and 7 days, Table S2. Relative abundance of identified bacterial isolates picked from MRS agar of the pork mince fermentation processes (samples of pork batters 1, 2, 3, 4, 5, and 6) at days 0 and 7, encompassing *Latilactobacillus sakei*, *Latilactobacillus curvatus*, *Lactiplantibacillus plantarum* (formely known as *Lactobacillus plantarum*), *Lactococcus lactis*, *Carnobacterium* spp., *Leuconostoc carnosum*, *Leuconostoc mesenteroides*, *Corynebacterium variabilis*, *Enterococcus faecium*, and *Pantoea agglomerans*, Table S3. Relative abundance of identified bacterial isolates picked from MSA of the pork mince fermentation processes (samples of pork batters 1, 2, 3, 4, 5, and 6) at days 0 and 7, encompassing *Staphylococcus xylosus*, *Staphylococcus equorum*, *Staphylococcus saprophyticus*, *Staphylococcus epidermidis*, *Staphylococcus vitulinus*, *Staphylococcus sciuri*, *Staphylococcus capitis*, *Staphylococcus haemolyticus*, *Macrococcus caseolyticus*, and *Kurthia* sp., Table S4: Relative abundance of identified bacterial isolates picked from RAPID’Entero agar of the pork mince fermentation processes (samples of pork batters 1, 2, 3, 4, 5, and 6) at days 0 and 7, encompassing *Serratia proteamaculans*, *Serratia liquefaciens*, *Hafnia alvei*, *Hafnia paralvei*, *Rahnella aquatilis*, *Klebsiella* spp., *Citrobacter* sp., *Lelliottia amnigena*, *Proteus vulgaris*, and *Pseudomonas* sp., Table S5. Alpha-diversity metrics based on the relative abundances of bacterial species found during pork mince fermentation processes (samples of pork batters 1, 2, 3, 4, 5, and 6), through (GTG)_5_-PCR fingerprinting of genomic DNA. The Simpson (D) and Pielou (Je) indexes were calculated for all samples to measure their diversity and evenness, respectively, Table S6. Water activity (a_w_) values obtained for the fermentation processes of less conventional meat types, incubated at 23°C for 0 and 7 days, Table S7. Relative abundance of identified bacterial isolates picked from MRS agar of less conventional meat fermentation processes (replicates 1 and 2) at days 0 and 7, encompassing *Latilactobacillus sakei*, *Latilactobacillus curvatus*, *Lactiplantibacillus plantarum*, *Lactococcus lactis*, *Carnobacterium* spp., *Leuconostoc carnosum*, *Corynebacterium variabilis*, *Weissella* sp., *Enterococcus faecium*, *Hafnia alvei*, and *Klebsiella* sp., Table S8. Relative abundance of identified bacterial isolates picked from MSA of less conventional meat fermentation processes (replicates 1 and 2) at days 0 and 7, encompassing *Staphylococcus xylosus*, *Staphylococcus equorum*, *Staphylococcus saprophyticus*, *Staphylococcus succinus*, *Staphylococcus pasteuri*, *Staphylococcus epidermidis*, *Staphylococcus warneri*, *Staphylococcus simulans*, *Staphylococcus hyicus*, *Staphylococcus aureus*, *Bacillus safensis*, *Macrococcus caseolyticus*, *Enterococcus hirae*, and *Klebsiella* sp., Table S9. Relative abundance of identified bacterial isolates picked from RAPID’Entero agar of less conventional meat fermentation processes (replicates 1 and 2) at days 0 and 7, encompassing *Serratia proteamaculans*, *Serratia liquefaciens*, *Hafnia alvei*, *Hafnia paralvei*, *Rahnella aquatilis*, *Klebsiella* sp., *Lelliottia amnigena*, *Citrobacter* sp., *Proteus vulgaris*, *Enterobacter* sp., and *Pseudomonas* sp., Table S10. Alpha-diversity metrics based on the relative abundances of bacterial species found during less conventional meat fermentation processes (replicates 1 and 2) at days 0 and 7, through (GTG)_5_-PCR fingerprinting of genomic DNA. The Simpson (D) and Pielou (Je) indexes were calculated for all samples to measure their diversity and evenness, respectively.
