# Supplementary material for: The Use of Less Conventional Meats or Meat with High pH Can Lead to the Growth of Undesirable Microorganisms during Natural Meat Fermentation
Source: Foods. 2020 Oct 1;9(10):1386. doi: 10.3390/foods9101386 (PMC7601081; doi:10.3390/foods9101386)
Supplement: Supplementary file 1 [file foods-09-01386-s001.zip › foods-924464-supplementary.docx]

Table S1. Water activity (a_w_) values of the pork mince fermentation samples of pork batters 1, 2, 3, 4, 5, and 6, incubated at 23°C for 0 and 7 days.

| Sample | Time (days) | | a_w_ |
| --- | --- | --- | --- |
| 1 | | 0 | 0.979 |
|  | | 7 | 0.973 |
| 2 | | 0 | 0.978 |
|  | | 7 | 0.974 |
| 3 | | 0 | 0.980 |
|  | | 7 | 0.974 |
| 4 | | 0 | 0.986 |
|  | | 7 | 0.972 |
| 5 | | 0 | 0.985 |
|  | | 7 | 0.975 |
| 6 | | 0 | 0.981 |
|  | | 7 | 0.973 |

Table S2. Relative abundance of identified bacterial isolates picked from MRS agar of the pork mince fermentation processes (samples of pork batters 1, 2, 3, 4, 5, and 6) at days 0 and 7, encompassing *Latilactobacillus sakei*, *Latilactobacillus curvatus*, *Lactiplantibacillus plantarum* (formely known as *Lactobacillus plantarum*), *Lactococcus lactis*, *Carnobacterium* spp., *Leuconostoc carnosum*, *Leuconostoc mesenteroides*, *Corynebacterium variabilis*, *Enterococcus faecium*, and *Pantoea agglomerans*.

| Sample | Time (days) | Species (relative abundance, %) | | | | | | | | | |
| --- | --- | --- | --- | --- | --- | --- | --- | --- | --- | --- | --- |
|  |  | *Latl. sakei* | *Latl. curvatus* | *Lpl. plantarum* | *L. lactis* | *Carnobacterium* spp. | *Leuc. carnosum* | *Leuc. mesenteroides* | *C. variabilis* | *E. faecium* | *P. agglomerans* |
| 1 | 0 | 18 | 39 | 0 | 0 | 42 | 0 | 0 | 0 | 0 | 1 |
|  | 7 | 33 | 67 | 0 | 0 | 0 | 0 | 0 | 0 | 0 | 0 |
| 2 | 0 | 33 | 25 | 0 | 0 | 35 | 0 | 0 | 0 | 7 | 0 |
|  | 7 | 40 | 60 | 0 | 0 | 0 | 0 | 0 | 0 | 0 | 0 |
| 3 | 0 | 50 | 21 | 0 | 0 | 29 | 0 | 0 | 0 | 0 | 0 |
|  | 7 | 43 | 57 | 0 | 0 | 0 | 0 | 0 | 0 | 0 | 0 |
| 4 | 0 | 54 | 10 | 0 | 0 | 20 | 8 | 0 | 8 | 0 | 0 |
|  | 7 | 71 | 15 | 5 | 0 | 0 | 7 | 0 | 0 | 0 | 0 |
| 5 | 0 | 57 | 14 | 0 | 0 | 29 | 0 | 0 | 0 | 0 | 0 |
|  | 7 | 90 | 10 | 0 | 0 | 0 | 0 | 0 | 0 | 0 | 0 |
| 6 | 0 | 66 | 5 | 0 | 16 | 13 | 0 | 0 | 0 | 0 | 0 |
|  | 7 | 92 | 0 | 0 | 6 | 0 | 0 | 2 | 0 | 0 | 0 |

Table S3. Relative abundance of identified bacterial isolates picked from MSA of the pork mince fermentation processes (samples of pork batters 1, 2, 3, 4, 5, and 6) at days 0 and 7, encompassing *Staphylococcus xylosus*, *Staphylococcus equorum*, *Staphylococcus saprophyticus*, *Staphylococcus epidermidis*, *Staphylococcus vitulinus*, *Staphylococcus sciuri*, *Staphylococcus capitis*, *Staphylococcus haemolyticus*, *Macrococcus caseolyticus*, and *Kurthia* sp..

| Sample | Time (days) | Species (relative abundance, %) | | | | | | | | |
| --- | --- | --- | --- | --- | --- | --- | --- | --- | --- | --- |
|  |  | *S. xylosus* | *S. equorum* | *S. saprophyticus* | *S. epidermidis* | *S. vitulinus* | *S. capitis* | *S. haemolyticus* | *M. caseolyticus* | *Kurthia sp.* |
| 1 | 0 | 18 | 52 | 20 | 1 | 2 | 2 | 0 | 5 | 0 |
|  | 7 | 23 | 47 | 20 | 0 | 0 | 0 | 0 | 10 | 0 |
| 2 | 0 | 25 | 35 | 35 | 0 | 0 | 0 | 0 | 2 | 0 |
|  | 7 | 15 | 70 | 10 | 0 | 0 | 0 | 5 | 0 | 0 |
| 3 | 0 | 35 | 34 | 28 | 3 | 0 | 0 | 0 | 0 | 0 |
|  | 7 | 40 | 35 | 22 | 3 | 0 | 0 | 0 | 0 | 0 |
| 4 | 0 | 23 | 7 | 70 | 0 | 0 | 0 | 0 | 0 | 0 |
|  | 7 | 18 | 29 | 45 | 0 | 0 | 0 | 0 | 0 | 2 |
| 5 | 0 | 21 | 28 | 51 | 0 | 0 | 0 | 0 | 0 | 0 |
|  | 7 | 10 | 40 | 50 | 0 | 0 | 0 | 0 | 0 | 0 |
| 6 | 0 | 0 | 15 | 85 | 0 | 0 | 0 | 0 | 0 | 0 |
|  | 7 | 0 | 30 | 70 | 0 | 0 | 0 | 0 | 0 | 0 |

Table S4: Relative abundance of identified bacterial isolates picked from RAPID’Entero agar of the pork mince fermentation processes (samples of pork batters 1, 2, 3, 4, 5, and 6) at days 0 and 7, encompassing Serratia proteamaculans, Serratia liquefaciens, Hafnia alvei, Hafnia paralvei, Rahnella aquatilis, Klebsiella spp., Citrobacter sp., Lelliottia amnigena, Proteus vulgaris, and Pseudomonas sp..

| Sample | Time (days) | Species (relative abundance, %) | | | | | | | | |  |
| --- | --- | --- | --- | --- | --- | --- | --- | --- | --- | --- | --- |
|  |  | *S. proteamaculans* | *S. liquefaciens* | *H. alvei* | *H. paralvei* | *R. aquatilis* | *Klebsiella* sp. | *Citrobacter* sp. | *L. amnigena* | *P. vulgaris* | *Pseudomonas sp.* |
| 1 | 0 | 23 | 26 | 12 | 0 | 23 | 3 | 0 | 10 | 3 | 0 |
|  | 7 | 10 | 10 | 70 | 0 | 0 | 0 | 10 | 0 | 0 | 0 |
| 2 | 0 | 0 | 20 | 30 | 0 | 0 | 44 | 6 | 0 | 0 | 0 |
|  | 7 | 0 | 30 | 40 | 0 | 0 | 0 | 30 | 0 | 0 | 0 |
| 3 | 0 | 0 | 20 | 20 | 0 | 20 | 0 | 30 | 0 | 0 | 10 |
|  | 7 | 0 | 70 | 15 | 0 | 0 | 15 | 0 | 0 | 0 | 0 |
| 4 | 0 | 10 | 25 | 40 | 0 | 0 | 25 | 0 | 0 | 0 | 0 |
|  | 7 | 0 | 87 | 3 | 0 | 0 | 10 | 0 | 0 | 0 | 0 |
| 5 | 0 | 20 | 0 | 15 | 0 | 20 | 0 | 10 | 0 | 0 | 35 |
|  | 7 | 0 | 75 | 15 | 10 | 0 | 0 | 0 | 0 | 0 | 0 |
| 6 | 0 | 0 | 10 | 10 | 0 | 40 | 10 | 0 | 0 | 0 | 30 |
|  | 7 | 0 | 80 | 20 | 0 | 0 | 0 | 0 | 0 | 0 | 0 |

Table S5. Alpha-diversity metrics based on the relative abundances of bacterial species found during pork mince fermentation processes (samples of pork batters 1, 2, 3, 4, 5, and 6), through (GTG)5-PCR fingerprinting of genomic DNA. The Simpson (D) and Pielou (Je) indexes were calculated for all samples to measure their diversity and evenness, respectively.

| Samples | Time (days) | Simpson (D) | Pielou (Je) |
| --- | --- | --- | --- |
| 1 | 0 | 0.89 | 0.32 |
|  | 7 | 0.84 | 0.37 |
| 2 | 0 | 0.89 | 0.36 |
|  | 7 | 0.85 | 0.39 |
| 3 | 0 | 0.89 | 0.37 |
|  | 7 | 0.85 | 0.39 |
| 4 | 0 | 0.87 | 0.35 |
|  | 7 | 0.81 | 0.34 |
| 5 | 0 | 0.87 | 0.38 |
|  | 7 | 0.80 | 0.38 |
| 6 | 0 | 0.81 | 0.35 |
|  | 7 | 0.77 | 0.39 |

Table S6. Water activity (a_w_) values obtained for the fermentation processes of less conventional meat types, incubated at 23°C for 0 and 7 days.

| Meat type | Time (days) | a_w_ | |
| --- | --- | --- | --- |
| Hare | 0 | | 0.979 |
|  | 7 | | 0.973 |
| Wild boar | 0 | | 0.978 |
|  | 7 | | 0.974 |
| Wild duck | 0 | | 0.980 |
|  | 7 | | 0.974 |
| Beef | 0 | | 0.986 |
|  | 7 | | 0.972 |
| Horse | 0 | | 0.985 |
|  | 7 | | 0.975 |
| Wild deer | 0 | | 0.981 |
|  | 7 | | 0.973 |

Table S7. Relative abundance of identified bacterial isolates picked from MRS agar of less conventional meat fermentation processes (replicates 1 and 2) at days 0 and 7, encompassing *Latilactobacillus sakei*, *Latilactobacillus curvatus*, *Lactiplantibacillus plantarum*, *Lactococcus lactis*, *Carnobacterium* spp., *Leuconostoc carnosum*, *Corynebacterium variabilis*, *Weissella* sp., *Enterococcus faecium*, *Hafnia alvei*, and *Klebsiella* sp..

| Meat type | Time (days) | Species (Relative abundance, %) | | | | | | | | |
| --- | --- | --- | --- | --- | --- | --- | --- | --- | --- | --- |
|  |  | *Latl. sakei* | *Latl. curvatus* | *Carnobacterium* spp. | *Leuc. carnosum* | *C. variabilis* | *Weissella* sp. | *E. faecium* | *H. alvei* | *Κlebsiella* sp. |
| Hare (1) | 0 | 60 | 10 | 20 | 0 | 0 | 0 | 0 | 0 | 0 |
|  | 7 | 95 | 5 | 0 | 0 | 0 | 0 | 0 | 0 | 0 |
| Hare (2) | 0 | 50 | 30 | 20 | 0 | 0 | 0 | 0 | 0 | 0 |
|  | 7 | 95 | 0 | 0 | 0 | 0 | 0 | 5 | 0 | 0 |
| Wild boar (1) | 0 | 60 | 10 | 20 | 0 | 10 | 0 | 0 | 0 | 0 |
|  | 7 | 95 | 0 | 0 | 0 | 0 | 0 | 0 | 5 | 0 |
| Wild boar (2) | 0 | 70 | 8 | 22 | 0 | 0 | 0 | 0 | 0 | 0 |
|  | 7 | 100 | 0 | 0 | 0 | 0 | 0 | 0 | 0 | 0 |
| Wild duck (1) | 0 | 60 | 30 | 10 | 0 | 0 | 0 | 0 | 0 | 0 |
|  | 7 | 100 | 0 | 0 | 0 | 0 | 0 | 0 | 0 | 0 |
| Wild duck (2) | 0 | 80 | 10 | 10 | 0 | 0 | 0 | 0 | 0 | 0 |
|  | 7 | 100 | 0 | 0 | 0 | 0 | 0 | 0 | 0 | 0 |
| Beef (1) | 0 | 35 | 30 | 35 | 0 | 0 | 0 | 0 | 0 | 0 |
|  | 7 | 90 | 5 | 0 | 0 | 0 | 0 | 5 | 0 | 0 |
| Beef (2) | 0 | 50 | 12 | 22 | 10 | 0 | 0 | 0 | 0 | 0 |
|  | 7 | 90 | 0 | 0 | 0 | 0 | 0 | 0 | 0 | 0 |
| Horse (1) | 0 | 90 | 10 | 0 | 0 | 0 | 0 | 0 | 0 | 0 |
|  | 7 | 100 | 0 | 0 | 0 | 0 | 0 | 0 | 0 | 0 |
| Horse (2) | 0 | 70 | 50 | 30 | 0 | 0 | 0 | 0 | 0 | 0 |
|  | 7 | 100 | 0 | 0 | 0 | 0 | 0 | 0 | 0 | 0 |
| Wild deer (1) | 0 | 70 | 10 | 0 | 0 | 20 | 0 | 0 | 0 | 0 |
|  | 7 | 95 | 0 | 0 | 0 | 5 | 0 | 0 | 0 | 0 |
| Wild deer (2) | 0 | 90 | 0 | 0 | 0 | 0 | 10 | 0 | 0 | 0 |
|  | 7 | 100 | 0 | 0 | 0 | 0 | 0 | 0 | 0 | 0 |

Table S8. Relative abundance of identified bacterial isolates picked from MSA of less conventional meat fermentation processes (replicates 1 and 2) at days 0 and 7, encompassing *Staphylococcus xylosus*, *Staphylococcus equorum*, *Staphylococcus saprophyticus*, *Staphylococcus succinus*, *Staphylococcus pasteuri*, *Staphylococcus epidermidis*, *Staphylococcus warneri*, *Staphylococcus simulans*, *Staphylococcus hyicus*, *Staphylococcus aureus*, *Bacillus safensis*, *Macrococcus caseolyticus*, *Enterococcus hirae*, and *Klebsiella* sp..

| Meat type | Time (days) | Species (Relative abundance, %) | | | | | | | | | | | | | |
| --- | --- | --- | --- | --- | --- | --- | --- | --- | --- | --- | --- | --- | --- | --- | --- |
|  |  | *S. xylosus* | *S. equorum* | *S. saprophyticus* | *S. succinus* | *S. pasteuri* | *S. epidermidis* | *S. warneri* | *S. simulans* | *S. hyicus* | *S. aureus* | *B. safensis* | *M. caseolyticus* | *E. hirae* | *Klebsiella* sp*.* |
| Hare (1) | 0 | 35 | 15 | 25 | 0 | 5 | 0 | 0 | 0 | 0 | 0 | 0 | 0 | 0 | 20 |
|  | 7 | 0 | 0 | 0 | 0 | 0 | 0 | 0 | 0 | 0 | 0 | 0 | 0 | 0 | 0 |
| Hare (2) | 0 | 20 | 60 | 10 | 0 | 0 | 10 | 0 | 0 | 0 | 0 | 0 | 0 | 0 | 0 |
|  | 7 | 0 | 0 | 0 | 0 | 0 | 0 | 0 | 0 | 0 | 0 | 0 | 0 | 100 | 0 |
| Wild boar (1) | 0 | 40 | 25 | 15 | 0 | 0 | 0 | 0 | 0 | 8 | 4 | 0 | 8 | 0 | 0 |
|  | 7 | 40 | 25 | 30 | 0 | 5 | 0 | 0 | 0 | 0 | 0 | 0 | 5 | 0 | 0 |
| Wild boar (2) | 0 | 30 | 45 | 20 | 0 | 0 | 5 | 0 | 0 | 0 | 0 | 0 | 0 | 0 | 0 |
|  | 7 | 50 | 20 | 20 | 0 | 0 | 10 | 0 | 0 | 0 | 0 | 0 | 0 | 0 | 0 |
| Wild duck (1) | 0 | 20 | 15 | 5 | 60 | 0 | 0 | 0 | 0 | 0 | 0 | 0 | 0 | 0 | 0 |
|  | 7 | 20 | 0 | 0 | 80 | 0 | 0 | 0 | 0 | 0 | 0 | 0 | 0 | 0 | 0 |
| Wild duck (2) | 0 | 10 | 10 | 5 | 45 | 0 | 10 | 20 | 0 | 0 | 0 | 0 | 0 | 0 | 0 |
|  | 7 | 20 | 10 | 0 | 55 | 0 | 0 | 10 | 0 | 0 | 5 | 0 | 0 | 0 | 0 |
| Beef (1) | 0 | 25 | 40 | 35 | 0 | 0 | 0 | 0 | 0 | 0 | 0 | 0 | 0 | 0 | 0 |
|  | 7 | 35 | 25 | 40 | 0 | 0 | 0 | 0 | 0 | 0 | 0 | 0 | 0 | 0 | 0 |
| Beef (2) | 0 | 5 | 50 | 45 | 0 | 0 | 0 | 0 | 0 | 0 | 0 | 0 | 0 | 0 | 0 |
|  | 7 | 20 | 45 | 30 | 0 | 0 | 0 | 0 | 5 | 0 | 0 | 0 | 0 | 0 | 0 |
| Horse (1) | 0 | 0 | 25 | 60 | 0 | 0 | 0 | 0 | 0 | 0 | 0 | 15 | 0 | 0 | 0 |
|  | 7 | 0 | 60 | 40 | 0 | 0 | 0 | 0 | 0 | 0 | 0 | 0 | 0 | 0 | 0 |
| Horse (2) | 0 | 5 | 45 | 50 | 0 | 0 | 0 | 0 | 0 | 0 | 0 | 0 | 0 | 0 | 0 |
|  | 7 | 0 | 60 | 40 | 0 | 0 | 0 | 0 | 0 | 0 | 0 | 0 | 0 | 0 | 0 |
| Wild deer (1) | 0 | 15 | 35 | 50 | 0 | 0 | 0 | 0 | 0 | 0 | 0 | 0 | 0 | 0 | 0 |
|  | 7 | 0 | 20 | 70 | 0 | 0 | 0 | 0 | 0 | 0 | 0 | 10 | 0 | 0 | 0 |
| Wild deer (2) | 0 | 5 | 30 | 65 | 0 | 0 | 0 | 0 | 0 | 0 | 0 | 0 | 0 | 0 | 0 |
|  | 7 | 0 | 35 | 65 | 0 | 0 | 0 | 0 | 0 | 0 | 0 | 0 | 0 | 0 | 0 |

Table S9. Relative abundance of identified bacterial isolates picked from RAPID’Entero agar of less conventional meat fermentation processes (replicates 1 and 2) at days 0 and 7, encompassing Serratia proteamaculans, Serratia liquefaciens, Hafnia alvei, Hafnia paralvei, Rahnella aquatilis, Klebsiella sp., Lelliottia amnigena, Citrobacter sp., Proteus vulgaris, Enterobacter sp., and Pseudomonas sp..

| Meat type | Time (days) | Species (Relative abundance, %) | | | | | | | | | | |
| --- | --- | --- | --- | --- | --- | --- | --- | --- | --- | --- | --- | --- |
|  |  | *S. proteamaculans* | *S. liquefaciens* | *H. alvei* | *H. paralvei* | *R. aquatilis* | *Klebsiella* sp. | *L. amnigena* | *Citrobacter* sp. | *P. bulgaris* | *Enterobacter* sp. | *Pseudomonas* sp. |
| Hare (1) | 0 | 10 | 0 | 10 | 0 | 0 | 80 | 0 | 0 | 0 | 0 | 0 |
|  | 7 | 0 | 0 | 40 | 0 | 0 | 20 | 0 | 0 | 0 | 40 | 0 |
| Hare (2) | 0 | 50 | 0 | 20 | 10 | 10 | 10 | 0 | 0 | 0 | 0 | 0 |
|  | 7 | 0 | 10 | 60 | 0 | 20 | 10 | 0 | 0 | 0 | 0 | 0 |
| Wild boar (1) | 0 | 5 | 0 | 20 | 10 | 35 | 5 | 0 | 20 | 0 | 0 | 5 |
|  | 7 | 10 | 0 | 55 | 10 | 0 | 0 | 0 | 20 | 5 | 0 | 0 |
| Wild boar (2) | 0 | 0 | 0 | 70 | 10 | 20 | 0 | 0 | 0 | 0 | 0 | 0 |
|  | 7 | 0 | 20 | 50 | 10 | 10 | 0 | 0 | 10 | 0 | 0 | 0 |
| Wild duck (1) | 0 | 10 | 0 | 20 | 0 | 60 | 0 | 0 | 0 | 0 | 0 | 10 |
|  | 7 | 5 | 0 | 70 | 20 | 0 | 0 | 0 | 0 | 5 | 0 | 10 |
| Wild duck (2) | 0 | 50 | 0 | 40 | 0 | 0 | 0 | 10 | 0 | 0 | 0 | 0 |
|  | 7 | 30 | 0 | 70 | 0 | 0 | 0 | 0 | 0 | 0 | 0 | 0 |
| Beef (1) | 0 | 70 | 10 | 20 | 0 | 0 | 0 | 0 | 0 | 0 | 0 | 0 |
|  | 7 | 95 | 0 | 5 | 0 | 0 | 0 | 0 | 0 | 0 | 0 | 0 |
| Beef (2) | 0 | 40 | 30 | 10 | 10 | 10 | 0 | 0 | 0 | 0 | 0 | 0 |
|  | 7 | 10 | 90 | 0 | 0 | 0 | 0 | 0 | 0 | 0 | 0 | 0 |
| Horse (1) | 0 | 0 | 0 | 60 | 9 | 10 | 21 | 0 | 0 | 0 | 0 | 0 |
|  | 7 | 0 | 10 | 70 | 10 | 5 | 0 | 0 | 5 | 0 | 0 | 0 |
| Horse (2) | 0 | 0 | 40 | 7 | 13 | 40 | 0 | 0 | 0 | 0 | 0 | 0 |
|  | 7 | 10 | 0 | 0 | 90 | 0 | 0 | 0 | 0 | 0 | 0 | 0 |
| Wild deer (1) | 0 | 10 | 0 | 50 | 10 | 20 | 0 | 10 | 0 | 0 | 0 | 0 |
|  | 7 | 0 | 0 | 80 | 10 | 0 | 0 | 10 | 0 | 0 | 0 | 0 |
| Wild deer (2) | 0 | 5 | 10 | 45 | 0 | 30 | 0 | 0 | 0 | 0 | 0 | 0 |
|  | 7 | 0 | 10 | 90 | 0 | 0 | 0 | 0 | 0 | 0 | 0 | 0 |

Table S10. Alpha-diversity metrics based on the relative abundances of bacterial species found during less conventional meat fermentation processes (replicates 1 and 2) at days 0 and 7, through (GTG)5-PCR fingerprinting of genomic DNA. The Simpson (D) and Pielou (Je) indexes were calculated for all samples to measure their diversity and evenness, respectively.

| Type of meat | Time (days) | Simpson | Pielou |
| --- | --- | --- | --- |
| Hare (1) | 0 | 0.82 | 0.36 |
|  | 7 | 0.68 | 0.42 |
| Hare (2) | 0 | 0.88 | 0.35 |
|  | 7 | 0.67 | 0.37 |
| Wild boar (1) | 0 | 0.90 | 0.32 |
|  | 7 | 0.82 | 0.34 |
| Wild boar (2) | 0 | 0.84 | 0.37 |
|  | 7 | 0.82 | 0.35 |
| Wild duck (1) | 0 | 0.86 | 0.36 |
|  | 7 | 0.77 | 0.37 |
| Wild duck | 0 | 0.85 | 0.34 |
|  | 7 | 0.78 | 0.38 |
| Beef (1) | 0 | 0.86 | 0.39 |
|  | 7 | 0.77 | 0.37 |
| Beef (2) | 0 | 0.87 | 0.36 |
|  | 7 | 0.78 | 0.38 |
| Horse (1) | 0 | 0.81 | 0.37 |
|  | 7 | 0.78 | 0.35 |
| Horse (2) | 0 | 0.85 | 0.39 |
|  | 7 | 0.74 | 0.46 |
| Wild deer (1) | 0 | 0.86 | 0.35 |
|  | 7 | 0.78 | 0.35 |
| Wild deer (2) | 0 | 0.80 | 0.37 |
|  | 7 | 0.74 | 0.46 |
